# The effects of fruit bagging on residue behavior and dietary risk for four pesticides in apple

**DOI:** 10.1038/s41598-018-32358-6

**Published:** 2018-09-25

**Authors:** Guofeng Xu, Jiyun Nie, Yonglong Wu, Zhen Yan, Mengliang Ye

**Affiliations:** Research Institute of Pomology, Chinese Academy of Agricultural Sciences/Laboratory of Quality & Safety Risk Assessment for Fruit (Xingcheng), Ministry of Agriculture, Xingcheng, 125100 P. R. China

## Abstract

In this study, the effects of fruit bagging on residue behavior and dietary risk for four pesticides (abamectin, imidacloprid, carbendazim and difenoconazole) in apple were investigated. The dissipation behavior of four pesticides were assessed after spraying on three occasions with the first spray at 2 months before harvest and the subsequent sprays at 10-day intervals at recommended doses of 5.4, 45, 135 and 975 g. a.i.ha^−1^, respectively. The dissipation experiment results demonstrated that apple fruit bagging reduced the initial deposits of four pesticides from 72.2% to 95.3%, prolonged the half-lives from 50.4% to 81.1%. The ultimate residues of abamectin, imidacloprid, carbendazim, and difenoconazole in bagged apple were far below the residues of unbagged apple. The dietary risks of four pesticides were assessed according to the ultimate residues and acceptable daily intakes (ADIs). The hazard quotient (HQ) were 0.013% to 43.415% for different pesticides. Fruit bagging reduced the HQ of four pesticides from 29.7% to 94.8%. Fruit bagging reduced the dietary risk of four pesticides in apple.

## Introduction

Apple is the second most consumed fruit in the world^1^. It contains many phenolic compounds which are beneficial to human health^2–4^. It is reported that phenolic can offer protection from cancer, cardiovascular conditions and some age-related diseases^5,6^. The nutritional value of apple is widely recognized, so the demand of apple is getting larger and larger. China is the largest country of apple production in the world. Apple planting has provided a significant contribution to increasing farmers’ incomes in apple producing areas. Fruit bagging is one of the important cultivation technique with multiple effects on inner quality^7,8^. It can improve apple quality and commercial value. The cultivation technique has been widely applied in China^9^. Fruit bagging is not only a physical protection technique against insect pests, birds, diseases, and mechanical scratches, but also alters the microenvironment on fruit development. The primary one is that fruit bagging can reduce levels of the light-absorptive compound anthocyanin^10^ and improve fruit coloration to increase their market value.

The pesticide is an indispensable material in apple production. It can reduce loss from pests and disease during the cultivation of apple, but it leads to serious agricultural environmental problems and the agricultural product quality safety problems. Abamectin, imidacloprid, carbendazim, and difenoconazole are widely applied in the control of apple tree pests and apple tree diseases in China. Abamectin and imidacloprid have touch-kill and stomach poison, low toxicity, photolytic fast, and can control spider mite^11^, aphid and leafhopper^12^ in apple orchards. Carbendazim and difenoconazole have good protective and treating effect, photolytic low and can used to control powdery mildew, anthracnose and gray mold^13^ in apple orchards. In order to protect human health and facilitate international trade, many countries and organizations have set the maximum residue limits (MRLs) of four pesticides in apple. At the same time, there are some reports on the dissipation of four pesticides in vegetables and fruits. Mahmoud *et al*.^14^ found that the half-life of abamectin was 1.02 days in strawberry. Under the conditions of 1.5 times recommended dosage, the half-lives were 2.1–3.4 days for carbendazim in tomato^15^. In recent study, the half-life of difenoconazole was 3.6 days following one time application in strawberry^16^. However, no studies have published on the residue behavior and dietary risk assessment of abamectin, imidacloprid, carbendazim, and difenoconazole in bagged apple.

In the present study, a UPLC-MS/MS method for the simultaneous determination of four pesticides (abamectin, imidacloprid, carbendazim, and difenoconazole) in apple was established and validated. The deposition and dissipation of four pesticides in unbagged apple and bagged apple were explored. Furthermore, the dietary risk from pesticide residues in apple was evaluated on the basis of consumption, acceptable daily intake (ADI) and the ultimate residues. The effects of fruit bagging on the residual characteristics of four pesticides in apple were investigated. This study may provide a scientific explanation for the effects of bagging on residue behavior and dietary risk assessment.

## Results

### Assay validation

The validation of the analytical method was evaluated by the following parameters: linearity, LOQ, matrix effect, recovery, precision and accuracy. The matrix calibration curves had better linear relationship in the range of 0.005–2.0 mg L^−1^, the correlation coefficients (*R*^2^) were 0.9931 to 0.9980 (Table 1). The limit of quantification (LOQ) was considered as the lowest concentrations with signal-to-noise ratio greater than 10. The matrix effect (ME) was evaluated by the percentage change of the slope ratio of the matrix-matched calibration and solvent calibration. As shown in Table 1, the LOQs were below 0.5 µg kg^−1^. The ME ranged from 22.6% to 66.1% and exceeded 15%, so matrix-matched standard solution was used to compensate for ME^17^. The accuracy was verified by measuring recovery for apple samples spiked at four different concentrations. Precision was assessed by performing five replicate measurements at each level. As shown in Table 2, the average recovery of four pesticides ranged from 81.7% to 99.5% for spiked concentration at 0.01, 0.05, 0.1 and 1 mg kg^−1^. The relative standard deviations ranged from 2.7% to 10.6%. The results were acceptable according to the National Standard of China^18^. The results indicated that the established method was sensitive and accurate for the determination of four pesticide residues in apple.

**Table 1:** Calibration equations, R^2^ values, LOQ values, and matrix effects for four pesticides.

Pesticides	Matrix	Regression equation	*R* ^2^	Matrix effect (%)	LOQ(µg kg^−1^)
Abamectin	ACN	y = 20.375x + 5.4827	0.9931	—	0.1
	Apple	y = 25.205x − 2.5329	0.9967	23.7	
Imidacloprid	ACN	y = 3.8372x + 13.694	099974	—	0.5
	Apple	y = 6.4125x + 17.208	0.9955	67.1	
Carbendazim	ACN	y = 93.63x + 371.51	0.9942	—	0.04
	Apple	y = 114.75x + 520.34	0.9967	22.6	
Difenoconazole	ACN	y = 3153.4x + 5876.6	0.9933	—	0.003
	Apple	y = 4425.5x + 4716.4	0.9980	40.3

**Table 2:** Recovery and the relative standard deviation (RSD) of four pesticides measurement in apple (n = 5).

Pesticides	0.01 mg kg^−1^		0.05 mg kg^−1^		0.1 mg kg^−1^		1.0 mg kg^−1^	
	Recovery (%)	RSD (%)	Recovery (%)	RSD (%)	Recovery (%)	RSD (%)	Recovery (%)	RSD (%)
Abamectin	85.2	10.6	89.2	4.9	92.3	3.3	87.6	6.3
Imidacloprid	81.7	4.6	83.2	8.1	88.6	3.4	93.9	3.1
Carbendazim	91.8	2.7	95.7	3.3	96.0	5.4	99.5	7.0
Difenoconazole	92.3	3.6	97.2	4.9	99.5	5.7	94.4	3.0

### The effect of fruit bagging on the initial deposits

Fruit bagging can not only protect apple from insect pests, birds, diseases, and mechanical scratches to a certain extent, it can also reduce direct contact between apples fruit and pesticides. By this way, fruit bagging can reduce the deposition of pesticide in apple fruit. In order to evaluate the effects of fruit bagging on four pesticides deposition, an experiment was performed in unbagged apple and bagged apple. As shown in Table 3, the initial deposits of abamectin, imidacloprid, carbendazim and difenoconazole in unbagged apple were 0.284, 0.502, 6.832 and 0.533 mg kg^−1^. In bag apple, the initial deposits of four pesticides were 0.079, 0.0237, 1.56 and 0.0588 mg kg^−1^. The initial deposits of abamectin, imidacloprid, carbendazim and difenoconazole in bagged apple had lost 72.2%, 95.3%, 77.2%, 89.0%, respectively. Fruit bagging can prevent most of pesticide from contaminating apple, so the initial deposits (2 h, day 0) of four pesticides in bagged apple are lower than the initial deposits of four pesticides in unbagged apple. In our experiment, the bag is double-layer bag and the inner of bag is red plastic material. Because of the red plastic has low permeability of water, so it can reduce direct contact between apple and pesticide to some extent. This may be the main reason why the initial deposits (2 h, day 0) of four pesticides in bagged apple are lower than in unbagged.

**Table 3:** The effect of apple fruit bagging on the initial deposits.

Pesticides	The initial deposits (mg kg^−1^)		The percentage reduction (%)
	Unbagged	bagged	
Abamectin	0.284	0.079	72.2
Imidacloprid	0.502	0.0237	95.3
Carbendazim	6.832	1.56	77.2
Difenoconazole	0.533	0.0588	89.0

### The effect of fruit bagging on degradation

The degradation trends of four pesticides in unbagged apple and bagged apple were shown in Table 4. The degradation of four pesticides were well described by the first-order kinetic (C_t_ = C_0_e^kt^) model in unbagged apple and bagged apple. The correlation coefficients (*R*^2^) were 0.9643 to 0.9940. The half-lives of abamectin, imidacloprid, carbendazim and difenoconazole in unbagged apple were 3.7, 7.0, 3.6, 12.1 days, respectively. The half-lives in bagged apple were 6.7, 10.8, 4.2 and 18.2 days, respectively. The half-lives in bagged apples were longer than in unbagged apples. The half-lives were prolonged 81.1%, 56.8%, 13.2%, 50.4%, respectively. Photolysis, hydrolysis and Microbial degradation of pesticide are the main degradation approaches^19,20^. Bag is made of the inner bag (red plastic) and the outer bag (yellow outside and black inside) in our experiment. Bag can prevent sunlight and water from reaching apple. Pesticides on the bagged apple surface expose in a dark and dry environment, so the photolysis and hydrolysis of pesticides are weak and the degradation of four pesticides are slow. At the same time, bag can influence the numbers and kinds of microbes on apple surface^21^. This can also lead to degrade more slowly.

**Table 4:** Dissipation dynamics equations, correlation coefficient (*r*) and half-life of four pesticides in unbagged apples and bagged apples.

Pesticides	Dissipation dynamics equation		*r*		Half- life/d	
	Unbagged	Bagged	Unbagged	Bagged	Unbagged	Bagged
Abamectin	C_t_ = 0.0276e^−0.187t^	C_t_ = 0.0070e^−0.103t^	0.9940	0.9830	3.7	6.7
Imidacloprid	C_t_ = 0.4373e^−0.099t^	C_t_ = 0.0198e^−0.064t^	0.9891	0.9662	7.0	10.8
Carbendazim	C_t_ = 5.9997e^−0.194t^	C_t_ = 0.9638e^−0.167t^	0.9940	0.9643	3.6	4.2
Difenoconazole	C_t_ = 0.4947e^−0.057t^	C_t_ = 0.0554e^−0.038t^	0.9938	0.9890	12.1	18.2

### The effect of apple fruit bagging on the ultimate residues

The ultimate residues of four pesticides in apple were presented in Table 5. 7 days after spraying pesticide, the residues of abamectin, imidacloprid, carbendazim, and difenoconazole in unbagged apple were 0.0077, 0.2010, 1.5300 and 0.3030 mg kg^−1^ and the residues in bagged apple were 0.0025, 0.0104, 0.1162 and 0.0409 mg kg^−1^, respectively. With the increased of DAS, the ultimate residues in bagged apple and unbagged apple were gradually decreased and were lower than the established MRLs in different countries and organizations^22–24^. Meanwhile, the ultimate residues of abamectin, imidacloprid, carbendazim, and difenoconazole in bagged apple were far below than in unbagged apple.

**Table 5:** Ultimate residues in apple for different treatments.

Pesticides	DAS 7d/mg kg^−1^		DAS 14d/mg kg^−1^		DAS 21d/mg kg^−1^		DAS 28d/mg kg^−1^	
	Unbagged	Bagged	Unbagged	Bagged	Unbagged	Bagged	Unbagged	Bagged
Abamectin	0.0077	0.0025	0.0027	0.0019	0.0008	0.00045	—	—
Imidacloprid	0.2010	0.0104	0.0800	0.0085	0.0570	0.0069	0.0320	0.0028
Carbendazim	1.5300	0.1162	0.2530	0.0756	0.1330	0.0489	0.0270	0.0084
Difenoconazole	0.3030	0.0409	0.2340	0.0348	0.1600	0.0227	0.0950	0.0204

DAS: Days after spraying; “—”: below LOQ.

### The effect of apple fruit bagging on the dietary risk

**Figure 1:**
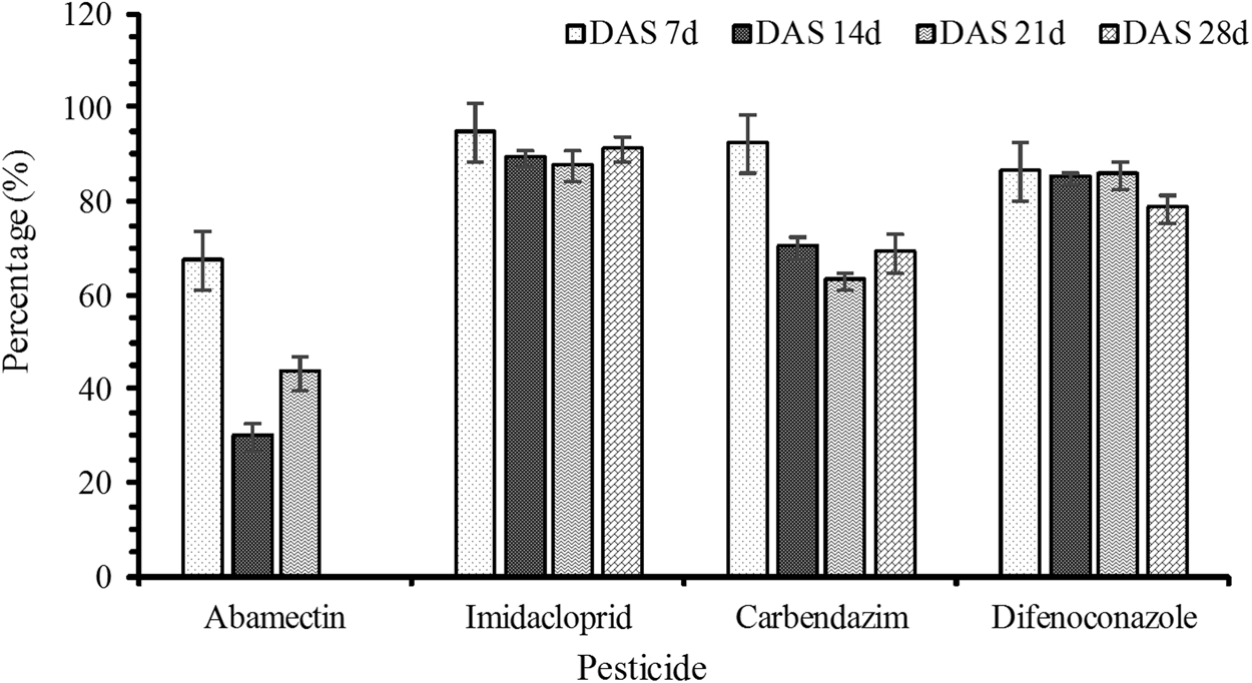
The effect of apple fruit bagging on the HQ.

The risk from pesticide residues in apple was evaluated through daily intakes (EDI) and acceptable daily intake (ADI). The average daily per capita consumption of apple was obtained from GEMS/Food Cluster Diets database^25^. Apple belongs to pome fruit fresh group, its consumption accounted for 2.11% (34.051 g day^−1^) of total daily food consumption (1612 g day^−1^). Exposure was expressed as HQ. The risk assessment of four pesticides in unbagged apple and bagged apple was presented in Table 6. Based on the residues at each DAS day, the intakes of four pesticides in unbagged apple and bagged apple were quite low than the ADI. The HQ of four pesticides in unbagged apple ranged from 0.013% to 43.415%. The HQ of four pesticides in bagged apple ranged from 0.023% to 8.598%. All the HQs were less than 100. At each DAS day, the HQ of each pesticide in bagged apple was lower than in unbagged apple. Fruit bag had reduced the HQ from 29.7% to 94.8%, of which abamectin was 29.6–67.5%, imidacloprid was 87.9–94.8%, carbendazim was 63.2–92.4% and difenoconazole was 78.5–86.5% (Fig. 1). The results showed that fruit bagging could effectively decrease the HQ of four pesticides in apple.

**Table 6:** The dietary risk assessment of four pesticides in unbagged apple and bagged apple.

Pesticides	ADI (mg/kg bw)	HQ (%)							
		DAS 7d		DAS 14d		DAS 21d		DAS 28d	
		Unbagged	Bagged	Unbagged	Bagged	Unbagged	Bagged	Unbagged	Bagged
Abamectin	0.002	0.218	0.071	0.077	0.054	0.023	0.013	—	—
Imidacloprid	0.06	5.704	0.295	2.270	0.241	1.617	0.196	0.908	0.079
Carbendazim	0.03	43.415	3.297	7.179	2.145	3.774	1.388	0.766	0.238
Difenoconazole	0.01	8.598	1.161	6.640	0.987	4.540	0.644	2.696	0.579

(Notice: The residues of abamectin were lower than LOQ in DAS 28d, so the HQ was blank).

## Discussion

LC-MS/MS is a very important detection method for pesticide residue^26^. In the present study, a UPLC-MS/MS method for determination of four pesticides (abamectin, imidacloprid, carbendazim, and difenoconazole) in apple was established. The linearity of matrix calibration curves had a good linear relation in the range of 0.005–2.0 mg L^−1^, the correlation coefficients (*R*^2^) were 0.993 to 0.998. At the lowest spiked level (0.01 mg kg^−1^), the LOQ were below 0.0005 mg kg^−1^. The matrix effect is usually regarded as a major drawback with LC—MS/MS in trace level analysis^27^. The ME of four pesticides in apple ranged 26.9% to 73.1% and exceeded 15%, so matrix-matched standard solution was used to compensate for ME^17^. For spiked concentrations between 0.01–1 mg kg^−1^, the average recovery of four pesticides ranged from 81.7% to 99.5% and the relative standard deviations ranged from 2.7% to 10.6%. The accuracy and precision of the method were acceptable according to the National Standard of China^18^.

Fruit bagging is one of the important cultivation methods in China^9^. It can reduce levels of the light-absorptive compound anthocyanin^10^ and improve fruit coloration, thus improve the apple quality and increase the commercial value of apple. Meanwhile, fruit bagging reduce pesticides on the apple surface, thus reduce the initial deposits and the ultimate residues of pesticide. At the recommended dosage and times, the initial deposits and the ultimate residues of abamectin, imidacloprid, carbendazim and difenoconazole in bagged apple were low than in unbagged apple (Tables 3 and 5). The results are consistent with the previous reports^28^. Fruit bagging can reduce pesticide residue in fruit.

Photolysis, hydrolysis and Microbial degradation of pesticide are the main degradation approaches^19,20^. The bagged apple was growing in almost dark and dry conditions, so the photolysis and hydrolysis of pesticides in bagged apple were weak. The degradation of four pesticides in bagged apple was slower than in unbagged apple, and the half-lives in bagged apple were long than unbagged apple (Table 3). At the same time, bagged could influence the numbers and kinds of microbes on apple surface^21^. It can also a factor that affect pesticide degradation.

The hazard quotient (HQ) is a method of the dietary risk assessment^26^. The HQ was evaluated on the basis of acceptable daily intake (ADI) for a 60-kg person and the estimated daily intakes (EDI). Our data demonstrated that the dietary exposure of abamectin, imidacloprid, carbendazim and difenoconazole for each DAS day were less than 100% in unbagged apple and bagged apple at the application of the recommended doses, but the HQ of each pesticide in bagged apple were lower than in unbagged apple. Fruit bagging can reduce the dietary risk of four pesticides in apple.

## Conclusions

In the present study, a method for the determination of abamectin, imidacloprid, carbendazim and difenoconazole in apple was established by using UPLC-MS/MS with modified QuEChERS extraction. The average recovery ranged from 81.7% to 99.5% and the relative standard deviations ranged from 2.7% to 10.6%. The LOQs were below 0.0005 mg kg^−1^. The four pesticides were applied to unbagged apple and bagged apple for thrice at the recommended dosage. The results suggested that fruit bagging reduced the initial deposits of four pesticides in apple from 72.2% to 95.3%. The half-lives of four pesticides were 3.7–12.1 days in unbagged apple and 4.3–18.2 days in bagged apple. The half-lives in bagged apple were prolonged 13.2–81.1%. At the recommend dosage, the ultimate residues of the pesticides in all apple samples were found to be below the MRLs of China (GB 2763, 2016), CAC (CAC, 2017) and European Commission (European Commission, 2015). The ultimate residues of four pesticides in bagged apple were lower than the residues in unbagged apple. The dietary exposure of four pesticides for each DAS day were less than 100% in unbagged apple and bagged apple, but the HQ of each pesticide in bagged apple were lower than in unbagged apple. Fruit bagging can reduce the dietary risk of four pesticides in apple.

## Materials and Methods

### Materials and reagents

The analytical standards, abamectin, imidacloprid, carbendazim, and difenoconazole were purchased from Dr. Ehrenstorfer GmbH (Augsburg, Germany). Abamectin (1.8% wettable powder) and imidacloprid (50% wettable powder) were purchased from Rongbang Chemical Co., Ltd. (Shandong, China). Carbendazim (50% wettable powder) was obtained from Fuda Agrochemical Co., Ltd. (Jiangsu, China). Difenoconazole (30% water dispersible granule) was purchased from Yijia Agrochemical Co., Ltd. (Shandong, China). Acetonitrile was HPLC grade (Fisher Scientific, USA). Analytical grade of sodium chloride (NaCl) and anhydrous MgSO_4_ were purchased from Beijing Chemical Company (Beijing, China). Ultra-pure water was prepared using a Milli-Q reagent water system (Bedford, MA, USA). Formic acid was obtained from the Tedia Company, Inc. (Fairfield, OH, USA). Primary secondary amine (PSA) was obtained from Angela Technologies Inc. (Tianjin, China). Kobayashi Bag KM-2 was purchased from Qingdao Kobayashi Bag Mfg. Co., Ltd. (Shandong, China).

Standard stock solution (100 mg/L) of abamectin, imidacloprid, carbendazim, and difenoconazole were prepared in pure ACN, respectively. Standard working solutions at 5, 20, 50, 100, 500, 1000, and 2000 μg/L concentrations were prepared from the stock solution by serial dilution. Correspondingly, matrix matched standard solutions of abamectin, imidacloprid, carbendazim, and difenoconazole were obtained at 5, 20, 50, 100, 500, 1000, and 2000 μg/L concentrations by adding blank apple sample extracts to each serially diluted standard solution. All solutions were protected against light with aluminum foil and stored in a refrigerator in the dark at −20 °C. The working standard solutions underwent no degradation for 3 months.

### Field experiment

The apple cultivars (*Malus domestica*), ‘Golden Delicious’ were used in this study. The trees were 8-year-old on *Malus micromalus* rootstocks. They were trained in a central leader system and grown at a 3.0 m × 4.0 m spacing and grown in northeast China (Xingcheng, 120.7°E, 40.6°N). In early june, about ninety well exposed fruits per tree were selected without bagging-treatment. Whereas the rest were bagged with light impermeable double layer (the outer layer is yellow outside and black inside, and the inner layer is red) paper bags and bags were placed onto fruitlets 4 to 6 weeks after petal fall. For the last two years, abamectin, imidacloprid, difenoconazole and carbendazim were not sprayed for disease and pest control. The dissipation experiment in unbagged apple and bagged apple were carried out in four adjacent field plots and the block design was a completely randomize. Each pesticide formulation was separately sprayed thrice with 10-day intervals and the first spraying was at 2 months before harvest. Pesticides were dissolved in water at recommended dose and sprayed with a SX-LK18C backpack sprayer. The untreated plots were sprayed with water as control. Each experiment plot was three trees and a buffer zone was used to separate between the plots of different treatments. The pesticides were applied under good agricultural practices (GAPs). Abamectin, imidacloprid, difenoconazole and carbendazim were sprayed at the rate of 5.4, 45, 135 and 975 g active ingredient (a.i.) ha^−1^ as a recommended dose. For each replication, 20 fruits were randomly taken in different positions. For the bagged fruits, they were harvested without taking off the bags to avoid exposure to light before sample collections. Apple samples were collected 2 h and 1, 3, 5, 7, 14, 21 and 28 days after the last application. The samples were collected and reduced to 500 g according to NY/T 788^18^. The unbagged apple samples and bagged apple samples (not including bags) were chopped and homogenized in an Ultra-Turrax homogenizer (IKA-Werke, Staufen, Germany). The samples were stored in dark at −20 °C for further analysis.

### Sample preparation procedure

Samples (10 ± 0.1 g) were weighed into a 50 mL teflon centrifuge tube. Then, 10 mL acetonitrile was added to extract pesticide. The tubes were capped and stirred for 5 min. 2 g NaCl and 4 g MgSO_4_ were added and vortexed vigorously for 2 min. The tubes were immediately centrifuged (5000 rpm) for 3 min and then 2 mL of the upper layer (acetonitrile) was transferred into a 5 mL centrifuge tube, which contained an amount of sorbent (30 mg PSA and 150 mg MgSO_4_). The extracts were vortexed for 1 min and subsequently centrifuged for 2 min at 5000 rpm. The supernatant was filtered by a 0.22 µm nylon syringe filter and transferred to an auto-sampler vial for the UPLC-MS/MS injection.

### Instrument conditions

The Waters UPLC–MS/MS system consisted of a Waters Acquity UPLC system and a Waters (Milford, MA, USA) Xevo TQD triple quadrupole tandem mass spectrometer equipped with an electrospray ionisation (ESI) source. A Waters Acquity UPLC HSS T3 column (2.1 × 100 mm, 1.8 μm–particle size, Milford, MA, USA) was used for the separation of abamectin, imidacloprid, carbendazim, and difenoconazole and maintained at 40 °C. The mobile phase was consisted of chromatography grade acetonitrile (A) and 0.2% formic acid in water (B). The gradient program was as follows: time 0–2.0 min, 5–95% A, 2.0–2.5 min, 95%–5% A, 2.5–4.0 min, 5% A. The flow rate was 0.4 mL min^−1^. The injection volume was 5 μL. The retention times of carbendazim, imidacloprid, difenoconazole, and abamectin were 1.58, 1.63, 2.74 and 3.86 min, respectively.

The multiple reaction monitoring (MRM) mode was operated for the target compounds. All parameters for MRM transitions, cone voltage and collision energy were optimized to obtain the highest sensitivity and resolution (Table 7). The capillary voltage was set at 2.0 kV, desolvation temperature were maintained at 500 °C. A cone gas flow of 50 L h^−1^ and desolvation gas flow of 800 L h^−1^ were used. The nebulizer gas was 99.99% nitrogen, and the collision gas was 99.99% argon.

**Table 7:** MS parameters for the analyze of the four pesticides.

Compound	MW	CV (V)	Quantification ion transition	CE1 (eV)	Confirmatory ion transition	CE2 (eV)
Abamectin	890.7	18	890.7 → 305.3	28	890.7 → 567.6	15
Imidacloprid	256.1	22	256.1 → 175.0	20	256.1 → 209.0	14
Carbendazim	192.1	28	192.1 → 132.1	28	192.1 → 160.1	18
Difenoconazole	406.0	35	406.0 → 251.0	26	406.0 → 337.0	17

### Method validation

The method was validated to evaluate the linear range, the limit of quantification (LOQ), matrix effect, accuracy and precision. A seven-point standard solution and matrix-matched standard solutions were prepared at the concentration of 5, 20, 50, 100, 500, 1000, and 2000 μg L^−1^ and analyzed to evaluate the linearity of the method. The limit of quantification (LOQ) was considered as the lowest concentrations with signal-to-noise ratio greater than 10. The presence of matrix components can affect the ionization of the target compounds when ESI is used, which leaded to the matrix effect. Matrix effects (ME) were evaluated through comparing the slopes of the calibration curves obtained in matrix and in solvent. ME was calculated as follows: matrix effect (ME%) = [(slope of calibration curves in matrix − slope of calibration curves in solvent)/slope of calibration curves in solvent] × 100%. The recovery assays were performed to investigate the accuracy and precision of the method. The recovery experiments were conducted by five replicates at four spiked levels (10, 50, 100 and 1000 μg kg^−1^). The accuracy and the precision in these conditions for repeatability were expressed as the average recovery and the relative standard deviation (RSD).

### Data calculation and analysis

The dissipation kinetics of carbendazim, imidacloprid, difenoconazole, and abamectin in unbagged apple and bagged apple were determined by plotting residue concentration against time. The rate equation was calculated from the first order rate equation: C_t_ = C_0_e^−kt^, where C_t_ represents the concentration of the pesticide residue at time t, C_0_ represents the initial concentration and k is the rate constant. The half-life (t_1/2_) was calculated from the k value for each experiment, t_1/2_ = ln 2/k.

The risk from these four pesticide residues in unbagged apple and bagged apple were evaluated based on the data obtained from field trial and the acceptable daily intake (ADI) for a 60-kg person. The estimated daily intakes (EDI) are calculated from the following equation^26^:

$${\rm{EDI}}={\rm{residue}}\,{\rm{amount}}\times {\rm{average}}\,{\rm{intake}}/60.$$

where residue amount is obtained from field trial, average intake is obtained from GEMS/Food Cluster Diets database, and 60 is standard weight for Chinese people. The dietary risk assessment is performed by using the following equations^29,30^:

$$HQ=EDI/ADI\times 100 \% .$$

where HQ is the hazard quotient, ADI (mg kg^−1^ bw^−1^) is the acceptable daily intake, and EDI (mg kg^−1^ bw^−1^) is the estimated daily intake. The risk is considered unacceptable when HQ was higher than 100%^31^.
